# Case Report: Conservative management of herpes simplex keratitis with corneal perforation and anterior chamber collapse

**DOI:** 10.3389/fmed.2026.1749225

**Published:** 2026-02-02

**Authors:** Wenjia Xie, Tian Tian, Luyi Ying

**Affiliations:** Department of Ophthalmology, Sir Run Run Shaw Hospital, Zhejiang University School of Medicine, Hangzhou, China

**Keywords:** anterior chamber collapse, case report, corneal perforation, herpes simplex keratitis, PCR

## Abstract

**Aim:**

This study aims to report the successful conservative management of herpes simplex keratitis (HSK) complicated by corneal perforation and anterior chamber collapse, emphasizing the importance of accurate diagnosis and close clinical monitoring to avoid surgical intervention.

**Methods:**

A 54-year-old male patient with recurrent redness and pain in the left eye was referred for potential surgical intervention due to corneal perforation and anterior chamber collapse. Examination revealed central corneal infiltrates, a positive Seidel test, and anterior chamber collapse. A tear specimen was collected for polymerase chain reaction (PCR) testing to detect viruses, including herpes simplex virus (HSV), varicella zoster virus (VZV), cytomegalovirus (CMV), human adenovirus (HAdV), and Epstein–Barr virus (EBV).

**Results:**

PCR testing confirmed herpes simplex virus infection in this patient. Treatment with oral valacyclovir, topical ofloxacin ointment, and 0.1% fluorometholone eye drops resulted in significant clinical improvement. Over the course of 1 month, visual acuity improved from counting fingers at 80 cm to 20/30, corneal lesions healed, and the anterior chamber reformed without the need for surgical intervention.

**Conclusion:**

This case demonstrates that conservative management can be effective for HSK with corneal perforation and anterior chamber collapse, particularly when supported by accurate diagnosis and close clinical monitoring.

## 1 Introduction

Herpes simplex keratitis (HSK) is one of the leading causes of infectious corneal blindness worldwide, characterized by recurrent episodes of corneal inflammation and scarring (1). The management of HSK is challenging due to its recurrent nature and the potential for severe complications such as corneal perforation (2). Precise and timely diagnosis of HSK is essential to initiate appropriate therapy and prevent further tissue damage (3). While oral antiviral therapy, particularly acyclovir and its prodrug valacyclovir, remains the cornerstone of treatment (1, 4), surgical interventions such as penetrating keratoplasty (PK) or deep anterior lamellar keratoplasty (DALK) may be necessary in cases where medical therapy fails, especially in patients with corneal perforation or anterior chamber collapse, to preserve the integrity of the globe (5–7).

However, performing keratoplasty in eyes with HSK is traditionally considered high risk and prone to graft failure (8). Patients with active inflammation, ulceration, and perforation due to HSK have particularly low surgical success rates; therefore, many surgeons avoid performing the procedure in such high-risk eyes during periods of active inflammation (9). DALK offers a higher success rate compared to PK due to a significantly reduced risk of endothelial rejection (5). However, it is technically more demanding, especially in perforated eyes, due to the high risk of intraoperative Descemet’s membrane rupture requiring conversion to PK (5).

This case report presents a patient with HSK complicated by corneal perforation and anterior chamber collapse. Contrary to the predominant advocacy for early surgical intervention, we attempted and successfully achieved conservative management with close monitoring, resulting in corneal healing and anterior chamber reformation. This case report highlights the role of polymerase chain reaction (PCR) in confirming the diagnosis and provides a new perspective on the indications for surgical intervention in such cases.

## 2 Case presentation

A 54-year-old male patient presented to our ophthalmology clinic with a 10-year history of recurrent redness and pain in the left eye, which had worsened over the preceding 3 days. The patient had intermittently used unspecified steroid eye drops for the past 10 years, without oral medication. The symptoms had occasionally improved but repeatedly recurred until a severe exacerbation occurred 3 days prior to presentation. He then visited a local hospital and was diagnosed with viral keratitis. He was prescribed oral acyclovir 200 mg five times daily, along with topical levofloxacin eye drops. One day prior to presentation, he accidentally touched the left eye, causing a sudden decline in vision. Upon re-evaluation at the local hospital, corneal perforation was detected. The patient received no further treatment there and was immediately referred to our institution for surgical consultation.

**Figure 1:**
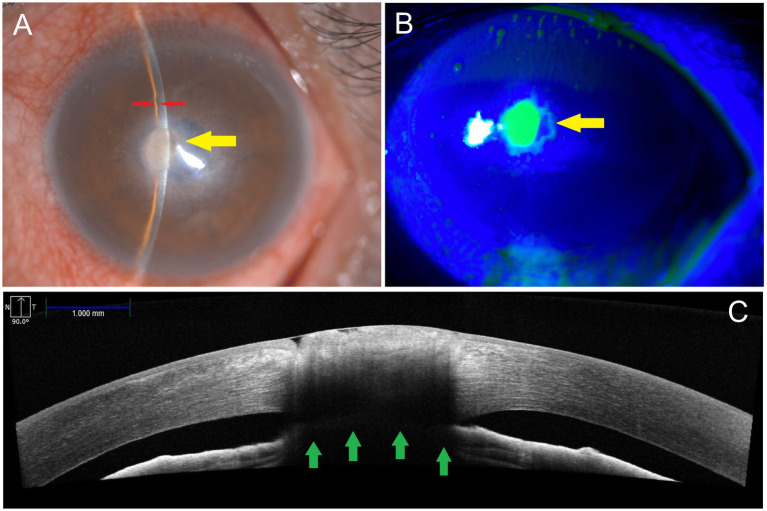
Initial presentation. **(A)** Slit lamp examination (Topcon slit lamp microscope) showed conjunctival injection, central corneal infiltrates, and corneal edema. **(B)** The Seidel test with fluorescein staining revealed a corneal perforation measuring 1.5 mm (vertical) by 1.0 mm (horizontal), located 1 mm nasal to the central visual axis (yellow arrows). **(C)** High-resolution anterior segment optical coherence tomography (Cirrus OCT, Carl Zeiss Meditec Inc.; axial resolution: 5 μm; transverse resolution: 15 μm; scan speed: 68000 A-scans per second) showed a corneal perforation with iris adhesion (green arrows). The anterior chamber was shallow (red arrows) on slit lamp examination but still visible on OCT.

Examination revealed a visual acuity of 20/20 in the right eye and counting fingers at 80 cm in the left eye. The right eye was unremarkable on slit lamp examination. Slit lamp examination of the left eye revealed conjunctival injection and central corneal infiltrates measuring 1.5 mm vertically by 1.0 mm horizontally, located 1 mm nasal to the central visual axis (Figure 1A). The Seidel test was positive, indicating a corneal perforation (Figure 1B). Corneal edema and layered scarring were observed around the perforation site, and the anterior chamber was collapsed. High-resolution anterior segment optical coherence tomography (OCT) confirmed the presence of a corneal perforation with iris adhesion at the perforation site. The anterior chamber was shallow but still present (Figure 1C).

The patient was previously healthy and denied any history of systemic diseases or manifestations of autoimmune or systemic inflammatory conditions, such as rheumatoid arthritis or atopic dermatitis.

**Figure 2:**
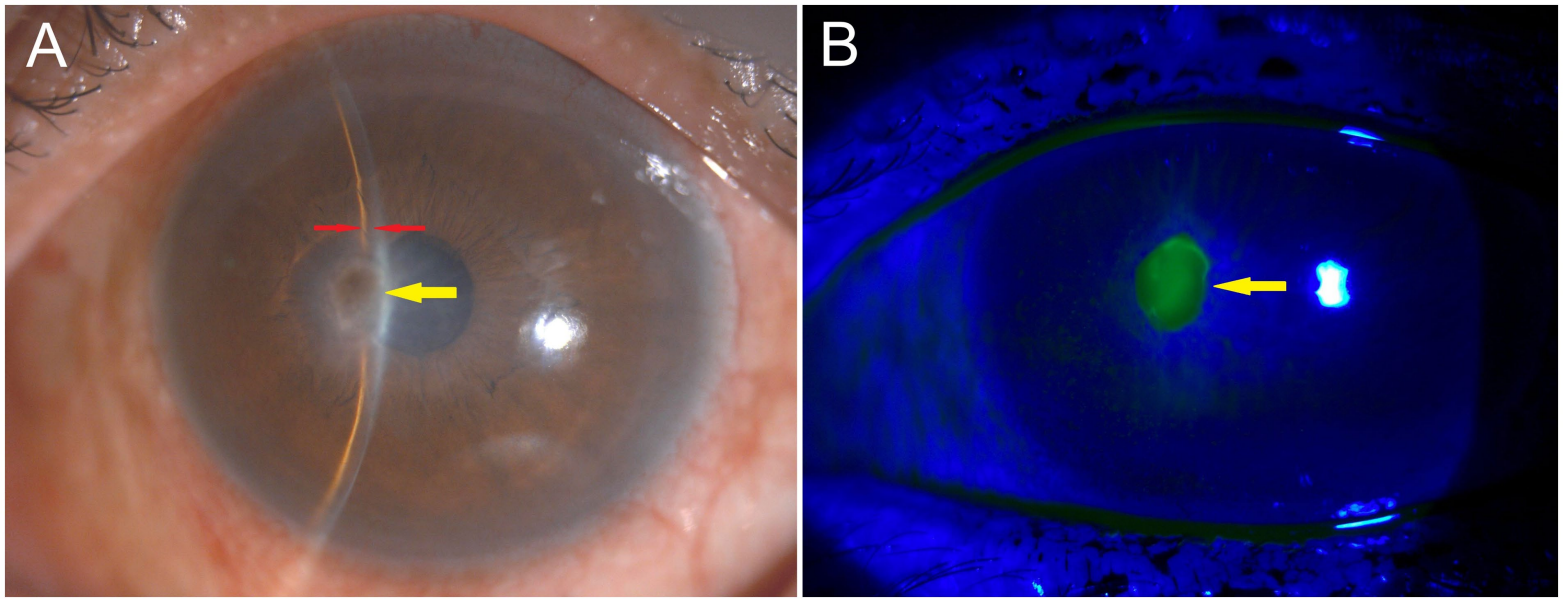
Three days after the initiation of treatment. **(A)** Slit lamp examination showed reduced conjunctival injection, decreased corneal infiltrates and edema (yellow arrows), and a slightly deeper anterior chamber (red arrows). **(B)** The Seidel test with fluorescein staining revealed minimal leakage.

A tear specimen was collected using a Schirmer test strip placed in the lower conjunctival fornix and sent for PCR testing to detect viruses that could infect the human body, including herpes simplex virus (HSV), varicella zoster virus (VZV), cytomegalovirus (CMV), human adenovirus (HAdV), and Epstein–Barr virus (EBV) (3). The patient was started on oral valacyclovir 1 g three times daily, topical ofloxacin ointment twice daily, and 0.1% fluorometholone eye drops four times daily. Three days after treatment, the patient’s visual acuity in the left eye improved to 20/200. Slit lamp examination (Figure 2A) showed reduced conjunctival injection, decreased corneal infiltrates and edema, a slightly deeper anterior chamber, and minimal leakage on Seidel testing (Figure 2B). The PCR results confirmed the presence of HSV, establishing the diagnosis of HSK.

**Figure 3:**
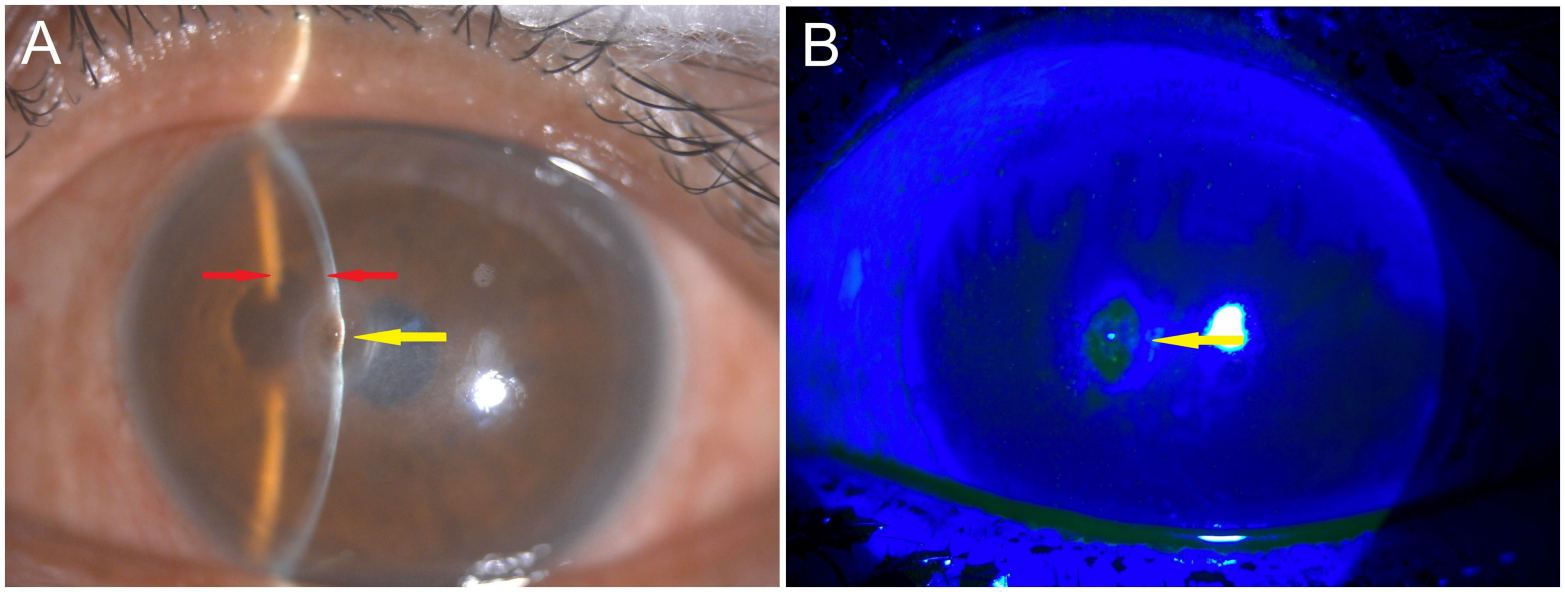
Seven days later (day 10 of treatment). **(A)** Slit lamp examination showed corneal lesion scarring (yellow arrows) and a fully reformed anterior chamber (red arrows). **(B)** The Seidel test with fluorescein staining was negative for leakage.

**Figure 4:**
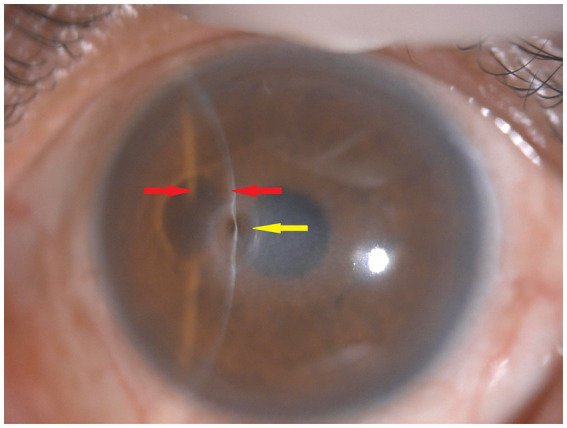
After 1 month of treatment, the corneal lesion was fully scarred with residual pigmentation (yellow arrows), and the anterior chamber remained deep (red arrows). Fluorescein staining was not performed.

Seven days later, the patient’s visual acuity further improved to 20/60. Slit lamp examination showed corneal lesion scarring and a fully reformed anterior chamber (Figure 3A). The Seidel test was negative for leakage (Figure 3B). After 1 month of treatment, the patient’s visual acuity improved to 20/30, the corneal lesion was fully scarred with residual pigmentation, and the anterior chamber remained deep (Figure 4). No corneal neovascularization was observed. The patient was subsequently lost to in-person follow-up. During a telephone consultation, the patient expressed satisfaction with the treatment outcome and wished to continue follow-up at a local hospital. We informed the patient that, having completed a 1-month therapeutic course of valacyclovir (1 g three times daily), he should now maintain the regimen of prophylactic-dose valacyclovir (500 mg once daily) and topical fluorometholone (four times daily) for additional weeks to complete at least 10 weeks of combined therapy (10). Due to his history of recurrent episodes, prophylactic valacyclovir was recommended for a minimum of 12 months (10). The local hospital was instructed to monitor for dendritic or geographic epithelial defects and to taper the 0.1% fluorometholone gradually according to the clinical response. Throughout the treatment course, intraocular pressure was not measured due to concerns about exacerbating the perforation. No signs of glaucoma were observed on fundus examination.

## 3 Discussion

This case illustrates the successful conservative management of HSK complicated by corneal perforation and anterior chamber collapse. At the onset of the current episode, the patient had already started treatment with oral acyclovir and topical antibiotic eye drops at the local hospital. However, the recommended therapeutic dose for HSV epithelial keratitis is acyclovir 400 mg 3–5 times daily (10). For more complex stromal keratitis with epithelial ulceration, a higher therapeutic dose of acyclovir 800 mg five times daily is recommended (10). Although it is unclear whether epithelial ulceration was present at the initial visit, the lack of topical corticosteroid use at the local hospital suggests that active viral replication may have been suspected or present. In such a scenario, the prescribed dose of 200 mg five times daily would likely have been subtherapeutic and might have contributed to insufficient viral suppression. The assessment of the initial treatment response was interrupted when the patient accidentally touched his left eye 2 days later, resulting in a sudden perforation. The subsequent clinical course demonstrates that adequate systemic antiviral therapy, combined with cautious use of mild corticosteroids to control inflammation, can lead to significant improvement even in severe cases of corneal perforation and anterior chamber collapse. Oral anti-viral agents are preferred over topical agents due to their better safety profile for long-term use and better corneal penetration, and stromal HSK typically requires a combination of antiviral therapy and topical corticosteroids. In cases of stromal HSK with epithelial ulceration, the use of a limited topical corticosteroid dose in combination with a therapeutic dose of oral antiviral agents is recommended by the HSV Keratitis Treatment Guideline (10). It is crucial to closely monitor for any signs of active viral replication such as dendritic or geographic epithelial defects. The balance between antiviral and corticosteroid therapy should be individualized depending on whether epithelial ulceration is present (4, 10).

A previous report of self-healing following corneal perforation demonstrated that an iris plug can provide mechanical closure for small corneal perforations and may promote rapid corneal healing by supplying blood to the avascular part of the cornea (11). In this case, a small portion of iris tissue adhered to the corneal perforation site due to anterior chamber collapse. Following corneal healing and reformation of the anterior chamber, the iris separated from the cornea, leaving residual pigmentation.

Close monitoring of the patient’s clinical course using slit lamp examination provided valuable insights into the progression or resolution of corneal perforation. The slit lamp findings, including improvement in the corneal lesion, Seidel test results, and anterior chamber depth were crucial for assessing the response to treatment. It is also important to remain vigilant for secondary complications, as glaucoma may develop due to persistent anterior chamber collapse or ongoing inflammation. Secondary bacterial infection is another factor that can complicate the management of corneal perforation. In this case, the use of topical ofloxacin ointment was effective in preventing bacterial superinfection.

The positive outcome in this case highlights important considerations regarding the role of conservative treatment versus surgical intervention, such as corneal transplantation, in similar scenarios. Although surgical management is often recommended for cases with anterior chamber collapse (6), this case suggests that conservative treatment may still be a viable option, particularly when the perforation is small, the infection is promptly controlled, and there is no evidence of secondary complications such as glaucoma. Although not used in this case, tissue adhesive glue and bandage contact lenses are additional treatment options for corneal perforations. For corneal perforations measuring approximately 1 mm, successful outcomes have been reported using an amniotic membrane transplant-plug technique combined with fibrin glue and a bandage contact lens (2, 12). A limitation of these methods, as well as of the present case, is that conservative management may be only feasible when the perforation is very small and the tissue plug successfully seals the defect, thereby creating a time window for healing. When the perforation is small, simple corneal suturing is also a potential treatment option. However, if the perforation is located within or near the visual axis, suturing may cause large corneal astigmatism and affect vision significantly. There is a reported case in which a single suture was used to secure a small corneal graft to repair a 1 mm perforation following corneal foreign body removal; this method could reduce corneal astigmatism (13). However, since suturing may exacerbate the inflammatory response, it is not suitable for perforations in the setting of active infection or inflammation. The decision to pursue surgical intervention should be guided by factors such as the size of the perforation, the extent of infection, the risk of secondary complications, and the patient’s clinical response to medical therapy (14).

The diagnosis of HSK is often based on slit lamp examination findings (15), but atypical clinical presentations of HSV infection can pose diagnostic challenges (16, 17). Molecular techniques such as PCR are valuable tools for confirming the presence of HSV in tear samples, guiding treatment decisions, and monitoring therapeutic response (17). In this case, the positive HSV PCR result confirmed the diagnosis of HSK and supported the continued use of valacyclovir. Given the recurrent nature of HSK, patients should be counseled on the importance of long-term antiviral prophylaxis to prevent future episodes. A limitation of our case report is that we did not measure corneal sensitivity and nerve distribution due to the lack of devices, which could have been helpful in both the diagnosis and the assessment of recovery in HSK.

## 4 Conclusion

In conclusion, this case report demonstrates that conservative management can still be a viable option for HSK complicated by corneal perforation and anterior chamber collapse, particularly when supported by accurate diagnosis and close monitoring. The successful outcome in this patient suggests that surgical intervention may not always be urgent, and conservative treatment should be considered a first-line approach in select cases. Further studies are needed to establish clear guidelines for the timing and indications of surgical interventions.

## Data Availability

The original contributions presented in the study are included in the article/supplementary material, further inquiries can be directed to the corresponding author.
